# Etiology and outcome of severe community acquired pneumonia in immunocompetent adults

**DOI:** 10.1186/1471-2334-13-94

**Published:** 2013-02-20

**Authors:** Ali Khawaja, Ali Bin Sarwar Zubairi, Fahad Khan Durrani, Afia Zafar

**Affiliations:** 1 Medical College, The Aga Khan University Hospital, PO Box # 3500, Stadium Road, Karachi, 74800, Pakistan; 2 Department of Medicine, Section of Pulmonary and Critical Care Medicine, The Aga Khan University Hospital, PO Box # 3500, Stadium Road, Karachi, 74800, Pakistan; 3 Department of Pathology and Microbiology, The Aga Khan University Hospital, PO Box # 3500, Stadium Road, Karachi, 74800, Pakistan

**Keywords:** Pneumonia, Severe community acquired pneumonia, Etiology, Outcome

## Abstract

**Background:**

Community Acquired Pneumonia (CAP) is a commonly encountered disease, one third of which is Severe Community Acquired Pneumonia (SCAP) that can be potentially fatal. There is a paucity of data on etiology and outcome of patients with SCAP in South Asian Population.

**Methods:**

A retrospective cross-sectional study was conducted from March 2002 till December 2008 on patients of 16 years and above who were admitted with the diagnosis of SCAP in accordance to the criteria of American Thoracic Society Guidelines (2001). The patients underwent clinical and diagnostic evaluations to detect the severity of illness as well as the etiology and other risk factors influencing the eventual outcome of SCAP.

**Results:**

A total of 189 patients were included in the study. The mean age was 60 ± 18.0 years and 110 (58%) patients were males. The most common isolated pathogens were *Staphylococcus aureus* (15 patients), *Streptococcus pneumoniae* (14 patients) and *Pseudomonas aeruginosa* (9 patients). The highest mortality was seen in patients with *Pseudomonas aeruginosa* (89%) and *Staphylococcus aureus* (53%). Overall mortality rate was 51%. On univariate analysis, septic shock (p <0.001), prior antibiotic use (p = 0.04), blood urea nitrogen > 30 mg/dl (p = 0.03), hematocrit < 30% (p = 0.03) and Acute Physiology and Chronic Health Evaluation (APACHE) II score > 20 (p < 0.001) were significantly different between the patients who survived as compared to those who did not. On multivariate analysis, septic shock (p <0.001, OR: 4.70; 95% CI= 2.49-8.87) was found to be independently associated with mortality.

**Conclusion:**

The microbes causing SCAP in our study are different from the usual spectrum. *Staphylococcus aureus* and *Pseudomonas aeruginosa* were the common causative pathogens and associated with high mortality. It is important to establish clinical guidelines for managing SCAP according to the etiologic organisms in our setting.

## Background

Community Acquired Pneumonia (CAP) is a frequently encountered lower respiratory tract parenchymal lung infection which continues to be a major health problem leading to significant morbidity and mortality worldwide [1]. The annual incidence of CAP varies from 5–11 per 1,000 population with the rates being higher in the elderly [1]. It presents a significant economic burden with the yearly cost amounting to US$12 billion [2]. The wide clinical spectrum of CAP varying from a mild self limiting infection to widespread sepsis leading to organ failure and death can be a daunting challenge for a physician to deal with.

Severe Community Acquired Pneumonia (SCAP) occurs in approximately 18-36% [3] of all CAP. The mortality rate for CAP is <5% for outpatient cases, it rises to 10% in admitted ward patients and can exceed 30% in patients admitted to intensive care unit (ICU) [4]. Tan *et al.* and Hirani *et al.* reported a mortality rate of 67% and 58% in patients with SCAP, respectively [3,5]. SCAP patients may require intensive care monitoring, mechanical ventilation and prolonged hospitalization, resulting in further economic burden especially in developing countries. Furthermore, despite the advancements in diagnosing and managing CAP in the past decades, the outcome remains unsatisfactory [5].

Although guidelines exist for the initial empirical treatment of SCAP [6,7], the emergence and spread of drug-resistant pathogens (including Penicillin-resistant *Streptococcus pneumoniae*), variations in the etiological agents, prevalence of atypical pathogens in different geographical locations and the seriousness of illness which demands initiation of treatment prior to reaching an etiologic diagnosis have led to ambiguities. It is hence imperative that these recommendations should be based on the epidemiological data obtained from a particular geographic location. There is paucity of data on SCAP in South Asia and the physicians tend to follow the American and British guidelines for managing this disease. Our study aimed to identify the etiological agents responsible for SCAP cases in a tertiary care hospital setting in Pakistan. We also assessed the factors influencing mortality and the actual outcome of patients with SCAP admitted in this setting.

## Methods

A retrospective cross-sectional study was conducted by the Section of Pulmonary and Critical Care Medicine at the Aga Khan University Hospital (AKUH), Karachi, from March 2002 till December 2008. AKUH is a major tertiary care hospital serving more than 10 million people of Karachi and the surrounding region. With an operational strength of 650 beds, the facility serves over 42,000 inpatients and over 500,000 outpatients annually. Established since 1985, it is one of the few teaching hospitals in South Asia accredited by the Joint Commission for International Accreditation [8].

All patients of age 16 years and above who were admitted with the diagnosis of SCAP in accordance to the criteria by American Thoracic Society guidelines (2001) were included in the study [6]. Patients who were transferred from another hospital and those who developed pneumonia after 48 hours of hospitalization or endotracheal intubation were excluded from the study to rule out hospital-acquired pneumonia. Patients less than 16 years of age and those who were immunocompromised (having underlying malignancy, undergoing chemotherapy or radiation, using high dose systemic corticosteroids and known HIV positive status) were also excluded from the study.

CAP was diagnosed on the basis of patient’s symptoms suggestive of pulmonary infection (fever, cough, sputum production, abnormal leukocyte count, pleuritic chest pain, signs of consolidation on examination) and chest x-ray finding of lung infiltrate. SCAP was defined as CAP associated with the presence of one major or two or more minor criteria. The major criteria included a need for mechanical ventilation and septic shock. The minor criteria included systolic BP ≤ 90 mmHg, bilateral pneumonia or multilobar pneumonia and PaO2/FIO2 ≤ 250.

The medical record numbers of patients are saved and coded according to the diagnoses at our institution. File records with primary diagnosis of pneumonia were extracted using the medical record numbers and reviewed. Imaging and laboratory software was used to review the radiology and laboratory and microbiological data. The recorded data included demographics of the patients including age, gender, comorbids, laboratory investigations including leukocyte count, platelet count, blood urea nitrogen and creatinine and arterial blood gases done on admission. Chest radiographs and cultures obtained within 48 hours of admission and outcome of the patients were evaluated. Chest radiographs were read by two experts separately and the mutual conclusions on the findings were obtained. The microbiological data comprised cultures of the respiratory tract (sputum, tracheal or endobronchial aspirate or bronchoalveolar lavage), acid fast bacilli smear and mycobacterial sputum cultures (in selected cases) and blood cultures. Serological testing for atypical bacteria and viruses was not performed due to non-availability of tests. The severity of illness was determined at the time of admission or transfer to the ICU within 48 hours of hospitalization. Mortality was defined as death of a patient during his or her hospital stay.

### Data analysis

The statistical analysis was conducted by using the Statistical package for social science SPSS (Release 16.0 standard version, copyright © SPSS). A descriptive analysis was performed for demographic and clinical characteristics and results are presented as mean ± standard deviation for quantitative variables and numbers (percentages) for qualitative variables. In univariate analysis, association between outcomes of SCAP and its risk factors was assessed by using the Chi-square test or Fisher exact test where appropriate. For contrasts of continuous variables, independent sample *t*-test was used to assess the difference of means. Univariable analyses were performed to examine the effect of each variable on the poor outcome. A forward stepwise selection method was used and variables significantly associated with the outcome of P < 0.05 were retained in the final model. To assess multivariate associations between the outcomes and potential covariates, odds ratios (ORs) and their 95% confidence intervals (CIs) were computed by logistic regression analysis.

### Ethical approval

Due to the retrospective nature of the study, a written informed consent could not be obtained from the participants. However, the study was done in compliance to International Helsinki Declaration and formal approval was taken from the Ethical Review Committee (ERC) of the Aga Khan University Hospital prior to commencement of the study. Identification of study participants was kept strictly confidential throughout the duration of the study.

## Results

### General characteristics of patients

A total of 832 files were reviewed out of which 189 patients met the inclusion criteria. The mean age was 60 ± 18.0 years and 110 (58%) patients were males. Twenty-nine (11%) patients had no co-morbid illnesses on presentation while the rest had at least one underlying disease which included cardiovascular disease (59%), pulmonary disease (42%) and diabetes mellitus (35%) (Table 1).

**Table 1:** Demographic, clinical, laboratory and radiographic characteristics of patients diagnosed with severe community acquired pneumonia (n = 189)

**Characteristics**	**Number of patients (percentage)**
**Age in years (Mean ± S.D.)**	60 ± 18.0
**Gender**	
Male	110 (58%)
Female	79 (42%)
**Underlying comorbids**	
Cardiovascular diseases	111 (59%)
Hypertension	92 (49%)
Ischemic heart disease	52 (28%)
Congestive heart failure	12 (06%)
Pulmonary diseases	79 (42%)
COPD	34 (18%)
Asthma	21 (11%)
Bronchiectasis	11 (06%)
History of Tuberculosis	10 (05%)
Pulmonary fibrosis	07 (04%)
Diabetes mellitus	66 (35%)
Others	52 (28%)
Chronic renal failure	41 (22%)
Chronic liver disease	11 (06%)
**Laboratory data**	
Hemoglobin (g/dL)	11.4 ± 2.7
Hematocrit (%)	34.2 ± 8.2
Total Leukocyte count (x 10^9^/L)	18.3 ± 13.5
Neutrophils (%)	82.0 ± 18.5
Lymphocytes (%)	11.4 ± 14.9
Plateletes (x 10^9^/L)	243 ± 138.5
Serum BUN (mg/dL)	41.0 ± 25.5
Serum Creatinine (mg/dL)	2.4 ± 2.2
Serum Sodium (mmol/L)	135.0 ± 7.4
Serum Potassium (mmol/L)	4.2 ± 0.9
Serum Bicarbonate (mmol/L)	20.0 ± 6.7
Serum Chloride (mmol/L)	102.0 ± 10.0
Arterial Blood Gases	
pH	7.36 ± 0.12
PaCO_2_ (mmHg)	38.0 ± 18.0
PaO_2_ (mmHg)	79.0 ± 26.0
**Chest radiography**	
Bilateral involvement	89 (47%)
Unilateral involvement (Right)	64 (34%)
Unilateral involvement (Left)	35 (19%)
Mediastinal involvement	01 (0.5%)
Consolidation	119 (63%)
Pleural effusion	70 (37%)
Interstitial infiltrates	53 (28%)
Atelectasis	30 (16%)
Cavitation	06 (03%)
Nodules	05 (03%)
Single lobe	123 (65%)
Multilobar	66 (35%)

### Microbial etiology of patients

Blood cultures were performed in all the patients while respiratory (sputum, tracheal aspirate, BAL) and pleural fluid cultures were available for 164 and 20 patients, respectively. Of 189 patients, 47 (25%) had an identifiable microbial etiology (Table 2). The most common isolated pathogens were *Staphylococcus aureus* (15 patients), *Streptococcus pneumoniae* (14 patients) and *Pseudomonas aeruginosa* (9 patients). *Mycobacterium tuberculosis* was diagnosed in 2 patients. *Nocardia asteroides* and *Streptococcus milleri* were found in one patient each; both organisms were isolated from pleural fluid culture. Besides the 100% mortality observed in three patients with *Moraxella catarrhalis, Burkholderia pseudomallei and Nocardia asteroids* respectively, the highest mortality was seen in patients with *Pseudomonas aeruginosa* (89%) and *Staphylococcus aureus* (53%).

**Table 2:** Isolation of bacteria from patients admitted with severe community acquired pneumonia (n = 189)

**Etiological agent**	**No. of patients with positive cultures**	**No. of patients who died**
*Staphylococcus aureus* (7 from blood culture, 5 from tracheal aspirate, 1 from BAL, 2 from sputum)	15	8 (53)
*Streptococcus pneumonia* (11 from blood culture, 1 from tracheal aspirate, 2 from sputum)	14	4 (29)
*Pseudomonas aeruginosa* (3 from blood culture, 3 from tracheal aspirate, 2 from BAL, 1 from sputum)	9	8 (89)
*Klebsiella pneumonia* (5 from tracheal aspirate, 1 from BAL)	6	3 (50)
*Mycobacterium tuberculosis* (2 from BAL)	2	1 (50)
*Moraxella catarrhalis* (Tracheal aspirate)	1	1 (100)
*Burkholderia pseudomallei* (Tracheal aspirate)	1	1 (100)
*Nocardia asteroids* (Pleural fluid)	1	1 (100)
*Streptococcus milleri* (Pleural fluid)	1	0
Number of pathogens isolated	50	27 (54)
Number of patients with known etiology*	47	25 (53)
Number of patients with unknown etiology	142	71 (50)

The values in the brackets are the percentages of patients who died with positive cultures.

*Three patients had dual etiologies: *Staphylococcus aureus* and *Pseudomonas aeruginosa* in two and *Pseudomonas aeruginosa* and *Nocardia asteroides* in one patient*.*

### Risk factors for mortality in patients

Chest radiography revealed bilateral lung involvement in 89 (47%), isolated right lung involvement in 64 (34%), isolated left lung involvement in 35 (19%) and mediastinal involvement in one patient. The most common finding was consolidation; 119 (63%) followed by pleural effusion in 70 (37%) and interstitial infiltrates in 53 (28%) patients (Table 1).

Out of 189 patients, 179 were admitted in ICU while 10 patients were managed in the wards. The average length of hospital stay was 10.2 ± 10.7 days with an average ICU and ward stay of 7 and 3 days, respectively. Eighty-four (44.4%) patients improved and were discharged home, 9 (4.7%) patients either left against medical advice or were shifted to another hospital due to non availability of ventilator machine and 96 (51%) patients died. Amongst all deaths, 59 (61%) occurred due to shock, 18 (19%) due to cardiac arrythmias, 17 (18%) due to respiratory failure and 2 (2%) were caused by multiorgan failure.

On univariate analysis, septic shock (p <0.001), prior antibiotic use in past 2 weeks (p = 0.04), blood urea nitrogen > 30 mg/dl (p = 0.03), hematocrit < 30% (p = 0.03) and Acute Physiology and Chronic Health Evaluation (APACHE) II score > 20 (p < 0.001) were statistically different between survivors and non-survivors (Table 3). After adjusting for significant variables in the univariate analysis, multivariate analysis revealed septic shock (p <0.001, OR: 4.70; 95% CI = 2.49-8.87) to be independently associated with mortality.

**Table 3:** Differences in clinical characteristics between survivors and non survivors (Univariate analysis)

**Clinical features**	**Survivors (n = 93)**	**Non survivors (n = 96)**	**p value**
**Demographic factors**			
Mean age	59.3 ± 18.6	61.0 ±17.4	0.52
≥ 65 years age	48 (52%)	52 (54%)	0.73
Gender male/female	55/38	55/41	0.79
**ATS major criteria**			
Use of mechanical ventilation	18 (19%)	29 (30%)	0.08
Septic shock	23 (25%)	59 (61%)	< 0.001
**History**			
Prior antibiotic use	14 (15%)	26 (27%)	0.04
Comorbids	76 (82%)	84 (88%)	0.27
**Physical examination findings**			
Blood pressure ≤ 90/60 mmHg	25 (27%)	30 (31%)	0.51
Pulse ≥ 120/min	32 (34%)	37 (39%)	0.55
Respiratory Rate 30/min	56 (60%)	57 (59%)	0.95
**Laboratory findings**			
Blood urea nitrogen ≥ 30 mg/dl	44 (47%)	59 (61%)	0.03
Creatinine > 1.5 mg/dL	48 (52%)	53 (55%)	0.25
Hematocrit <30%	22 (24%)	37 (39%)	0.03
Na <130 mmol/L	22 (24%)	28 (29%)	0.39
Arterial pH <7.35	28 (30%)	32 (33%)	0.63
**Radiographic findings**			
Pleural effusion	32 (34%)	38 (40%)	0.46
Bilateral involvement	36 (39%)	53 (55%)	0.07
Multilobar involvement	28 (30%)	38 (40%)	0.22
**Mean APACHE II score**	19.1 ±6.0	28.2 ±7.0	<0.001
**APACHE II score ≥ 20**	38 (41%)	80 (83.0%)	<0.001

## Discussion

Several studies have published clinical and epidemiological data on CAP, but very few have reported the data on SCAP, particularly in South Asian population. In this study, a special emphasis was given to identify the common etiological agents and outcomes associated with SCAP.

The diagnostic yield of 25% in this study was low in comparison to other studies of SCAP [9-12]. The most plausible explanations for the low yield is the non-availability and high cost of serological tests essential for the identification of atypical organisms like *Mycoplasma pneumoniae, Legionella pneumophila* and *Chlamydia pneumoniae* and frequent use of antibiotic in the community*.* Atypical pathogens have been the major cause of CAP in some studies from the west with *Legionella pneumophila* type 1 causing 1-30% [5,13,14] of adult cases and *Mycoplasma pneumoniae* being implicated in 20-30% [15,16] as an etiological agent.

*Streptococcus pneumoniae* was found to be the second most common pathogen accounting for pneumonia in around 7% of the patients. This percentage is lower when compared to the western countries where *Streptococcus pneumoniae* is reported in 24-45% of the patients with CAP and SCAP [5,9-14]. The most crucial aspect of our study was a high proportion of *Staphylococcus aureus and Pseudomonas aeruginosa* as the causative agents. Generally, these organisms are more associated with hospital acquired infection and less commonly reported for community acquired pneumonia [11,17]. However, amongst studies from the west, Pachon *et al.* reported *gram negative bacilli* in 25% of patients diagnosed with SCAP [13]. Ruiz *et al.* reported 4 out of 47 patients with known etiology to be infected with *Pseudomonas aeruginosa* in Spain [14] while a more recent study in the same region found 8 out of 39 patients to have *Pseudomonas aeruginosa* pneumonia in ICU patients [18]. Another study in Russia found *Staphylococcal aureus* and *Pseudomonas aeruginosa* to be important etiological agents in SCAP [19]. On the contrary in the Asian continent, the most common organisms isolated in patients from India were *Pseudomonas aeruginosa* (35%) and *Staphylococcus aureus* (24%) [20]. A relatively higher prevalence of *Pseudomonas aeruginosa* and *Staphylococcus aureus* has also been reported in Japan and China [21-24] indicating microbiological spectrum specific to certain epidemiological areas. Wu *et al.* reported around 23% of the patients to have *Pseudomonas aeruginosa* while another study in Japan reported 17% and 11% of the patients to have *Staphylococcal aureus* and *Pseudomonas aeruginosa* associated SCAP, respectively [23,24]. We found only two cases of pulmonary tuberculosis leading to SCAP in contrast to a study from Singapore in which 16% of the patients were diagnosed to have pulmonary tuberculosis [3]. Other studies in Asia particularly Singapore and Thailand have highlighted the presence of *Burkholderia pseudomallei* (3, 9) which is not prevalent in our setting. *Hemophilus influenzae* and *Moraxella catarrhalis* were also not isolated, possibly due to their inability to cause severe disease.

The physical examination and chest radiography findings did not vary significantly between the patients who survived and those who did not. Although higher number of deaths was observed in patients who required mechanical ventilation and those with bilateral involvement of lungs, these were not statistically significant. APACHE II score was significantly increased in non-survivors on univariate analysis. History of prior antibiotic use in last 2 weeks, BUN ≥ 30 mg/dl and hematocrit < 30% were significantly associated with high mortality on univariate analysis. Prior antibiotic use particularly in last 3 months is a well identified risk factor for colonization and infection associated with multi-drug resistant (MDR) pathogens [25,26]. However, on multivariate analysis, septic shock was the only variable independently associated with mortality in our study.

The overall mortality of patients in our study was lower than those reported by other Asian studies (3, 22), comparable to a recent Japanese study (21) and higher than western studies (10–14). However, low mortality was also seen in a study from Thailand [9] while a high mortality was observed in United Kingdom [5]. This reflects the wide variability that exists between SCAP outcomes in different settings and geographical regions. In our study, infection with *Pseudomonas aeruginosa* was associated with worst outcome as 8 out of 9 patients infected with this pathogen died. *Staphylococcus aureus* was also associated with a high mortality rate of 53%. In contrast to a study by Moine *et al.* in France where *Streptococcus pneumoniae* was significantly associated with mortality (35% patients) [12], it was relatively less fatal in our study. *Klebsiella pneumoniae* pneumonia which is common in European countries and highly associated with alcoholism and mortality [11] was also less common in our setting, possibly due to limited use of alcohol. The mortality in patients with identified etiology was almost the same as in patients in which no organism was isolated. The overall higher mortality could also be attributed to other factors like lack of national health care services, poverty, decreased level of awareness and health related myths and practices in general population.

The major limitation of our study was the lack of availability of serological tests for the diagnosis of viral and atypical pathogens. Furthermore, no set protocol was followed for the identification of microbial etiology. Investigations were done mainly on the discretion of the primary physician. Due to low percentage of patients with etiological identification, risk factors of patients with *Staphylococcus aureus* and *Pseudomonas aeruginosa* infection could not be ascertained. Risk stratification scores such as Pneumonia Severity Index (PSI) could not be calculated due to retrospective collection of data. Prospective trials would be required to further assess the indicators of severity and management of disease including time to admission to ICU, time to antimicrobial treatment, diagnosis of bacteremia and whether initial antimicrobial treatment was appropriate. Lastly, this was a single center study and hence, cannot be generalized to the whole population.

## Conclusion

In conclusion, the microbial spectrum causing SCAP found in this study varies considerably from the west. *Staphylococcus aureus* and *Pseudomonas aeruginosa* were amongst the most common causative pathogens and highly associated with mortality. This highlights the importance of a development of local pneumonia guidelines focusing on its etiology and management.

## Abbreviations

CAP: Community Acquired Pneumonia; SCAP: Severe Community Acquired Pneumonia; ATS: American Thoracic Society; APACHE: Acute physiology and chronic health evaluation; ICU: Intensive care unit; AKUH: Aga Khan University Hospital; BUN: Blood urea nitrogen; BAL: Bronchoalveolar lavage; OR: Odds ratio; CI: Confidence interval; ERC: Ethical Review Committee; MDR: Multi drug resistant.

## Competing interests

The authors declare no conflict of interest.

## Authors’ contributions

AK contributed in data entry, analysis and manuscript writing. ABSZ contributed in terms of original idea, study design, protocol writing and critically editing the manuscript. FKD contributed in data collection, data entry and manuscript writing. AZ reviewed the microbiological data and critically revised the manuscript. All authors have read and approve the final version of the manuscript.

## Pre-publication history

The pre-publication history for this paper can be accessed here:

http://www.biomedcentral.com/1471-2334/13/94/prepub

## References

[B1] Brar NK Niederman MS Management of community-acquired pneumonia: a review and update Ther Adv Respir Dis 2011 5 61 78 10.1177/1753465810381518 20935033

[B2] Colice GL Morley MA Asche C Birnbaum HG Treatment costs of community-acquired pneumonia in an employed population Chest 2004 125 2140 2145 10.1378/chest.125.6.2140 15189934

[B3] Tan YK Khoo KL Chin SP Ong YY Aetiology and outcome of severe community-acquired pneumonia in Singapore Eur Respir J 1998 12 113 115 10.1183/09031936.98.12010113 9701424

[B4] Nair GB Niederman MS Community-acquired pneumonia: an unfinished battle Med Clin North Am 2011 95 1143 1161 Nov 10.1016/j.mcna.2011.08.007 22032432 PMC7127066

[B5] Hirani NA Macfarlane JT Impact of management guidelines on the outcome of severe community acquired pneumonia Thorax 1997 52 17 21 10.1136/thx.52.1.17 9039234 PMC1758402

[B6] Niederman MS Mandell LA Anzueto A Bass JB Broughton WA Campbell GD Dean N File T Fine MJ Gross PA Martinez F Marrie TJ Plouffe JF Ramirez J Sarosi GA Torres A Wilson R Yu VL American Thoracic Society Guidelines for the management of adults with community-acquired pneumonia. Diagnosis, assessment of severity, antimicrobial therapy, and prevention Am J Respir Crit Care Med 2001 163 1730 1754 11401897 10.1164/ajrccm.163.7.at1010

[B7] Mandell LA Wunderink RG Anzueto A Bartlett JG Campbell GD Dean NC Dowell SF File TM Jr Musher DM Niederman MS Torres A Whitney CG Infectious Diseases Society of America American Thoracic Society Infectious diseases society of America/American thoracic society consensus guidelines on the management of community-acquired pneumonia in adults Clin Infect Dis 2007 44 Suppl 2 S27 S72 17278083 10.1086/511159 PMC7107997

[B8] Tariq M Jafri W Ansari T Awan S Ali F Shah M Jamil S Riaz M Shafqat S Medical mortality in Pakistan: experience at a tertiary care hospital Postgrad Med J 2009 85 470 474 10.1136/pgmj.2008.074898 19734514

[B9] Reechaipichitkul W Pisprasert V Severe community-acquired pneumonia (CAP) treated at Srinagarind Hospital, Khon Kaen, Thailand Southeast Asian J Trop Med Public Health 2004 35 430 433 15691151

[B10] Rello J Bodi M Mariscal D Navarro M Diaz E Gallego M Valles J Microbiological testing and outcome of patients with severe community-acquired pneumonia Chest 2003 123 174 180 10.1378/chest.123.1.174 12527619

[B11] Paganin F Lilienthal F Bourdin A Lugagne N Tixier F Génin R Yvin JL Severe community-acquired pneumonia: assessment of microbial aetiology as mortality factor Eur Respir J 2004 24 779 785 10.1183/09031936.04.00119503 15516672

[B12] Moine P Vercken JB Chevret S Chastang C Gajdos P Severe community-acquired pneumonia. Etiology, epidemiology, and prognosis factors Chest 1994 105 1487 1495 10.1378/chest.105.5.1487 8181342

[B13] Pachon J Prados MD Capote F Cuello JA Garnacho J Verano A Severe community-acquired pneumonia Am Rev Respir Dis 1990 142 869 873 10.1164/ajrccm/142.2.369 2382902

[B14] Ruiz M Ewig S Torres A Arancibia F Marco F Mensa J Sanchez M Martinez JA Severe community-acquired pneumonia. Risk factors and follow-up epidemiology Am J Respir Crit Care Med 1999 160 923 929 10471620 10.1164/ajrccm.160.3.9901107

[B15] Mansel K Rosenow EC Smith TF Martin JW Mycoplasma pneurnoniae pneumonia Chest 1989 95 639 4 10.1378/chest.95.3.639 2646077

[B16] Howard LS Sillis M Pasteur MC Kamath AV Harrison BD Microbiological profile of community-acquired pneumonia in adults over the last 20 years J Infect 2005 50 107 113 10.1016/j.jinf.2004.05.003 15667910

[B17] Almirall J Mesalles E Klamburg J Parra O Agudo A Prognostic factors of pneumonia requiring admission to the intensive care unit Chest 1995 107 511 516 10.1378/chest.107.2.511 7842786

[B18] Cillóniz C Ewig S Ferrer M Polverino E Gabarrús A Puig de la Bellacasa J Mensa J Torres A Community-acquired polymicrobial pneumonia in the intensive care unit: aetiology and prognosis Crit Care 2011 15 R209 10.1186/cc10444 21914220 PMC3334753

[B19] Sinopalnikov AI Tartakovskił IS Mironov MB Community-acquired pneumonia: Etiological diagnosis Antibiot Khimioter 1997 42 38 42 9412402

[B20] Shah BA Singh G Naik MA Dhobi GN Bacteriological and clinical profile of Community acquired pneumonia in hospitalized patients Lung India 2010 27 54 57 10.4103/0970-2113.63606 20616935 PMC2893425

[B21] Yoshimoto A Nakamura H Fujimura M Nakao S Severe community-acquired pneumonia in an intensive care unit: risk factors for mortality Intern Med 2005 44 710 716 10.2169/internalmedicine.44.710 16093592

[B22] Hu HC Huang CC Tsai YH Lee CH Hsieh MJ Outcome analysis of patients requiring mechanical ventilation with severe community-acquired pneumonia and identified bacterial pathogens Chang Gung Med J 2005 28 229 236 16013342

[B23] Wu CL Chan MC Chang GC Lee YL Chin CS Chang KM Hsu JY Etiology and cytokine expression in patients requiring mechanical ventilation due to severe community-acquired pneumonia J Formos Med Assoc 2006 105 49 55 10.1016/S0929-6646(09)60108-X 16440070

[B24] Kawai S Ochi M Nakagawa T Goto H Antimicrobial therapy in community-acquired pneumonia among emergency patients in a university hospital in Japan J Infect Chemother 2004 10 352 358 10.1007/s10156-004-0350-2 15614461

[B25] Kollef MH Micek ST Strategies to prevent antimicrobial resistance in the intensive care unit Crit Care Med 2005 33 1845 1853 10.1097/01.CCM.0000171849.04952.79 16096464

[B26] Barbosa TM Levy SB The impact of antibiotic use on resistance development and persistence Drug Resist Updat 2000 3 303 311 10.1054/drup.2000.0167 11498398

